# Temporal segmentation of EEG based on functional connectivity network structure

**DOI:** 10.1038/s41598-023-49891-8

**Published:** 2023-12-19

**Authors:** Zhongming Xu, Shaohua Tang, Chuancai Liu, Qiankun Zhang, Heng Gu, Xiaoli Li, Zengru Di, Zheng Li

**Affiliations:** 1 The International Academic Center of Complex Systems, Beijing Normal University, Zhuhai, 519087 China; 2 The Center for Cognition and Neuroergonomics, State Key Laboratory of Cognitive Neuroscience and Learning, Beijing Normal University, Zhuhai, 519087 China; 3 The School of Systems Science, Beijing Normal University, Beijing, 100875 China; 4 The State Key Laboratory of Cognitive Neuroscience and Learning, Beijing Normal University, Beijing, 100875 China

**Keywords:** Data processing, Network topology, Signal processing, Cognitive neuroscience, Complex networks

## Abstract

In the study of brain functional connectivity networks, it is assumed that a network is built from a data window in which activity is stationary. However, brain activity is non-stationary over sufficiently large time periods. Addressing the analysis electroencephalograph (EEG) data, we propose a data segmentation method based on functional connectivity network structure. The goal of segmentation is to ensure that within a window of analysis, there is similar network structure. We designed an intuitive and flexible graph distance measure to quantify the difference in network structure between two analysis windows. This measure is modular: a variety of node importance indices can be plugged into it. We use a reference window versus sliding window comparison approach to detect changes, as indicated by outliers in the distribution of graph distance values. Performance of our segmentation method was tested in simulated EEG data and real EEG data from a drone piloting experiment (using correlation or phase-locking value as the functional connectivity strength metric). We compared our method under various node importance measures and against matrix-based dissimilarity metrics that use singular value decomposition on the connectivity matrix. The results show the graph distance approach worked better than matrix-based approaches; graph distance based on partial node centrality was most sensitive to network structural changes, especially when connectivity matrix values change little. The proposed method provides EEG data segmentation tailored for detecting changes in terms of functional connectivity networks. Our study provides a new perspective on EEG segmentation, one that is based on functional connectivity network structure differences.

## Introduction

Neural activity involves information exchanges via connections at the cellular scale to the cortical scale. A powerful approach to understanding neural activity is analyzing the information exchanges, quantified via metrics on relatedness of neural data time series called functional connectivity measures. Particularly, analyzing the graphs (functional connectivity networks) built from neural data using complex network theory offers new methods for understanding and decoding brain activity.

Electroencephalograph (EEG) is a widely used neural activity recording method, and there are many EEG-based studies on functional connectivity networks^1–8^. The typical abstraction assumes a recording electrode or channel (corresponding roughly to a brain region) is a network node. Synchronized oscillations (coherence or correlation) between channels indicate the strength of edges (functional connections between brain regions). In these studies, functional connectivity networks are constructed and then analyzed to examine the relationship between different mental states, actions, or sensations and functional connectivity network structure. For example, by assessing network statistics at the global (network topology) and local (interregional connectivity) levels, Parkinson’s patients have been shown to have decreased functional connectivity and loss of frontotemporal connectivity with cognitive deterioration^6^. Mean cluster coefficients of both theta (4–7 Hz) and alpha (8–13 Hz) bands are lower when eyes are open during resting state versus when eyes are closed, and local efficiency is lower^7^. Music perception shows higher functional connectivity and enhanced small-world network organization compared to listening to noisy and silent backgrounds^8^.

In functional connectivity network analysis, a key assumption is the stationarity of the signal within the analysis scope^9^. Due to switching among inherent metastable states of neural assemblies during brain function and different time scales involved in the dynamical processes of cognition, EEG signals are non-stationary^10^. Here, non-stationarity means that the signal’s statistical characteristics change over time. Detecting the times at which changes occur is a critical issue to functional connectivity analysis: we must carefully set the window start and stop times to ensure stationarity within a connectivity analysis window so that connectivity metrics do not “blend” patterns from two or more qualitatively different functional networks. Thus, careful segmentation of EEG signals is a necessary step before analyses. Segmentation methods for EEG data have been proposed, which divide recorded data into time segments wherein statistics of data (thus hopefully brain function) are quasi-quiescent to a certain degree.

There currently exist many methods for EEG segmentation. Generally, they can be divided into two categories: single-channel and multi-channel. Many methods exist for single-channel segmentation. For example, when we assume that data belonging to the same segment is subject to a time-invariant model, the statistical properties of the model fitting error can be used as the criterion for segmenting^11^. There are other methods that segment based on statistical metrics^12,13^: boundaries are declared when test metrics significantly change. Multi-channel methods are needed to examine stationarity in terms of connectivity and interacting brain regions.

Existing methods based on multiple channels target different problems and statistical properties. One approach segments EEG based on structure as captured by the tensor decomposition^14^. A microstate analysis method based on functional connectivity graphs has been proposed^15^. Some studies have proposed features of functional connectivity for segmentation of electrophysiological data. They use analytical methods such as hidden Markov model (HMM), stochastic variational inference, and multi-dynamic adversarial generator-encoder (MAGE) to determine recurring brain states^16–22^. Another method^23^ detects time points at which there are changes in the spatial distribution of EEG voltage. Although there are several multi-channel segmentation methods and derivative methods, there are currently few segmentation methods specifically designed to detect functional connectivity network structure changes. In the analysis of brain functional connectivity networks, network structure reflects brain states mainly through indices that quantify node centrality, community structure, and connection density. As a precursor to these analyses, it is more straight-forward to segment EEG based on network structure directly via tests on these indices. To this end, we propose a method for segmenting based on these network structure features. Our network-based temporal segmentation approach has some similarities with the EEG microstate approach. Both segment EEG based on brain activity patterns; the EEG microstate approach segments based on matching to the predefined limited number of repeating brain states, and can be seen as an example of a multi-channel segmentation approach. We here segment based on functional connectivity network structural changes.

We here aim to build an EEG segmentation method which detects changes in functional connectivity on the graph structure level, using measures (indices) on graph structure as the basis of change detection. We aim to be agnostic to the functional connectivity metric used to build the graph, and we aim to allow different graph structure measures to be plugged into our framework, providing flexibility for different applications. Our contribution is an EEG segmentation method that looks for statistically significant changes in graph structure measures computed at each node (node importance indices, see Table 1). We evaluate our method on simulated data and real EEG data.

To quantify the difference in network structure between two windows of data, we propose an intuitive and flexible graph distance framework and use a sliding window comparison procedure to test if distance changes are significant (see “Sliding window change detection”). One of six node importance indices can be plugged into this graph distance framework. Different node importance indices examine different properties of networks. We compare our method to methods which segment based on distances computed not from graph structure, but from functional connectivity matrices directly (see “Comparison algorithms”).

To test our segmentation method, we simulated data wherein known network structure changes occur (see “Simulation results”). We also apply our method on real EEG data (see “Real EEG Data Results”), where connectivity was calculated by Pearson correlation or phase-locking value (PLV), though our method can be used with other connectivity metrics. Since we do not know the ground truth boundaries in the real data, we design an index to measure the segmentation quality and compare our method with alternatives. In the simulations and analysis on real data, we assume nodes in the network are EEG electrodes, but our segmentation method is equally applicable to source connectivity networks where nodes are voxels or regions of interest. The results show that, at least for correlation and PLV functional connectivity, our method produces better segmentation results than segmenting based on connectivity matrices directly, but the best performing node importance index has a high computational cost. We discuss our work, including parameter settings, similarities with other work, and limitations, in the “Discussion” and conclude in the “Conclusion”.

## Methods

### Goal of segmentation

**Figure 1:**
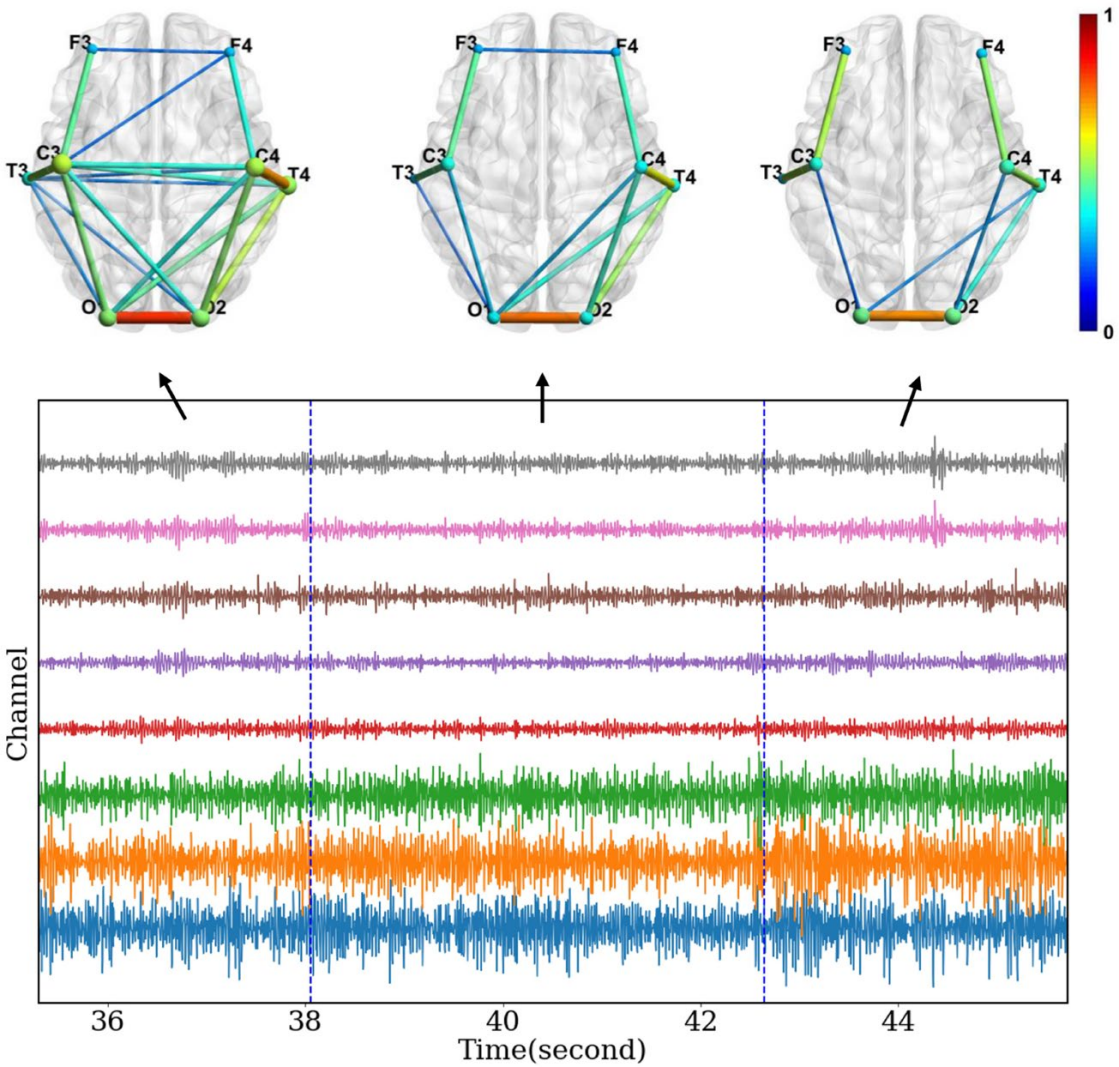
Example segmentation detected via our method (DMDC feature, edges from correlation). 8-channel EEG data (lower panel) and functional connections of three segments (upper panels). Only edges above 0.2 are shown to improve visualization. Size and color of the nodes indicate nodes’ degree centrality measure. Color of edges represents Pearson correlation coefficient(absolute value), which is unitless.

The goal of segmentation is to divide a time series into many intervals. Within each interval, the signals have similar statistical characteristics and can be deemed quasi-stationary. In this work, we design a segmentation method to detect time points at which there are changes in the network structure calculated from EEG, so that different time segments have different brain functional connectivity networks, while the network is stable within a time segment. Figure 1 is an example segmentation on real 8-channel data with three clearly different networks. The example in Fig. 1 comes from data used in our real EEG experiment. As described in more detail in “Real EEG Data Results”, this data comes from a mental workload experiment where participants controlled a virtual drone in a flying task with two mental workload levels. The original intent of the experiment was to collect data for developing a mental workload (high-low) classifier, and our research group is interested in applying functional connectivity network analysis on that data to generate better classification features. As a necessary step, we here develop a method for segmenting EEG data based on functional connectivity changes.

### Proposed algorithm

#### Functional connectivity network construction

**Figure 2:**
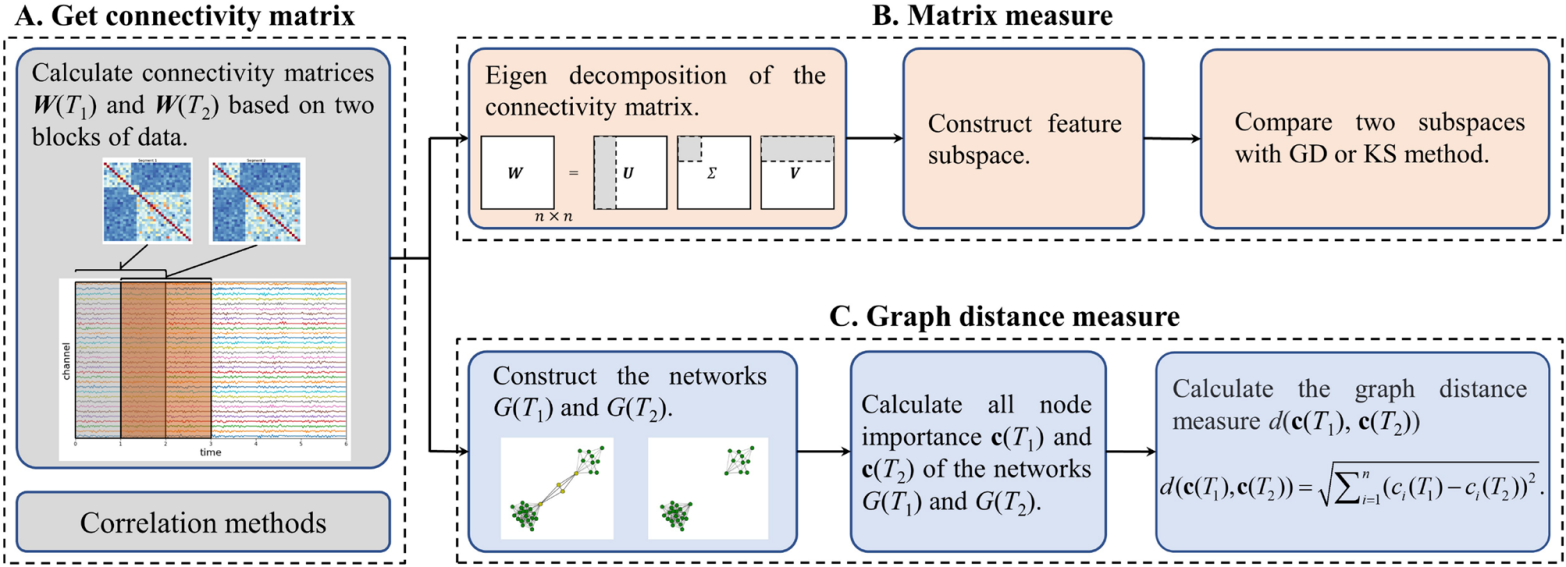
Flowchart of procedure for calculating graph distance measures (proposed method) and matrix measures (comparison method). (**A**) Generating connectivity matrices from two windows of EEG data. (**B**) Comparing connectivity matrices directly (comparison method). (**C**) Comparing networks using Euclidean distance on node importance index values (proposed method).

Given a segment of EEG data, the network connectivity matrix first needs to be calculated (Fig. 2A). Many connectivity metrics have been proposed, and among them commonly-used ones include: Pearson correlation coefficient (PC)^24^, mutual information^25,26^, phase-locking value (PLV)^27,28^, and phase lag index (PLI)^28^. We here use PC and PLV, though our segmentation method is agnostic to the particular choice of connectivity metric, and the merits of choices are not the focus of our study. A functional connectivity network is then constructed based on the connectivity matrix. The nodes represent brain regions surrounding corresponding EEG electrodes and edges represent the strength of communication between brain regions. When using a threshold on connection strength, the connectivity matrix becomes an adjacency matrix and the network becomes an unweighted network. A weighted network can be constructed if not using a threshold and keeping the original connection strengths. We work with undirected weighted networks in this study, so as to retain more information.

#### Computing distance between graphs

Graph distance measures (or graph similarity) describe the dissimilarity (similarity) between networks, quantifying topological differences between networks. Graph distance measures have long been a focus in network science and are widely used in many fields, such as pattern recognition^29^, model selection^30^, network classification and clustering^31^, anomaly, and discontinuity detection^32^. Previously proposed measures include^33–37^. We here build an intuitive and modular framework for graph distance by calculating a vector to summarize each graph and then computing distances between vectors representing different graphs. The vectors contain a value per node, where the value is an index of the node’s importance. This framework allows different node importance indices to be plugged in, resulting in different graph distance measures (abbreviations with the DM prefix).

Given two time periods $T_1$ and $T_2$ and EEG data with *n* channels, connectivity matrices $W(T_1)$ and $W(T_2)$ are calculated from the time periods, respectively. Weighted networks $G(T_1)$ and $G(T_2)$ are constructed from the connectivity matrices. The indices of node importance in networks $G(T_1)$ and $G(T_2)$ are represented by *n*-length vectors $\textbf{c}(T_1) = (c_{1}(T_1), c_{2}(T_1), \ldots ,c_{n}(T_1))$ and $\textbf{c}(T_2) = (c_{1}(T_2), c_{2}(T_2), \ldots ,c_{n}(T_2))$. We calculate graph distance $d(\textbf{c}(T_1), \textbf{c}(T_2))$ using the Euclidean distance between two node importance vectors, as follows:

1 $$\begin{aligned} d(\textbf{c}(T_1), \textbf{c}(T_2)) = \sqrt{\sum \nolimits _{i = 1}^n {{{({c_{i}(T_1)} - {c_{i}(T_2)})}^2}} }, \end{aligned}$$

We here consider six indices of node importance for weighted networks^38^, summarized in Table 1. DC, CC, EC, BC, CCO, and NNC denote degree centrality, closeness centrality, eigenvector centrality, betweenness centrality, clustering coefficient, and nearest neighbor centrality, respectively. Applying Eq. (1) to each node importance index gives each of the six corresponding graph distance measures: DMDC, DMCC, DMEC, DMBC, DMCCO, and DMNNC, respectively. These six indices are briefly compared in the “Discussion”. A schematic of the procedure for calculating graph distance is shown in Figure 2C.

**Table 1:** Definitions of node importance indices. Abbreviations in parentheses are names given to distance measures based on these node importance indices.

Index	Definition
Degree centrality (DMDC)	$DC_i = \frac{1}{n-1} \sum \limits _{j \in N_i} w_{ij}$ .
Closeness centrality (DMCC)	$CC_i = (\frac{1}{n-1} \sum \limits _{j \in N, j\ne i}^n {d_{ij}})^{-1}.$
Eigenvector centrality (DMEC)	$EC_i = \lambda ^{-1} \sum \limits _{j=1}^n w_{ij}x_{j}.$
Betweenness centrality (DMBC)	$BC_i = \frac{2}{(n-1)(n-2)} \sum \limits _{k \ne i \ne j} {\frac{g_{kij}}{g_{kj}}}$.
Clustering coefficient (DMCCO)	$CCO_i = \frac{\sum \limits _{ k \ne i} \sum \limits _{ j \ne i, j \ne k} w_{ij} w_{ik} w_{jk}}{\sum \limits _{ k \ne i} \sum \limits _{ j \ne i, j \ne k} w_{ij} w_{ik}}$.
Nearest neighbor centrality (DMNNC)	$NNC_i = \frac{1}{k_i - 1} \sum \limits _{j \in N_i} (n - 1)DC_j$.

${{\textbf {W}}}$ is the connectivity matrix of the network with elements $w_{ij}$. *n* is the number of nodes. $N_i$ is the set of all nodes connected to node *i*. *N* is the set of all nodes in the network. $k_i$ is the number of the set $N_i$. $d_{ij}$ is the length of the shortest path between node *i* and *j*. $g_{kj}$ is the number of shortest paths from node *k* to node *j*, and $g_{kij}$ is the number of shortest paths that pass through node *i* from node *k* to node *j*. $x_i$ is the eigenvector of the max eigenvalue $\lambda$ of matrix ${{\textbf {W}}}$.

#### Sliding window change detection

**Figure 3:**
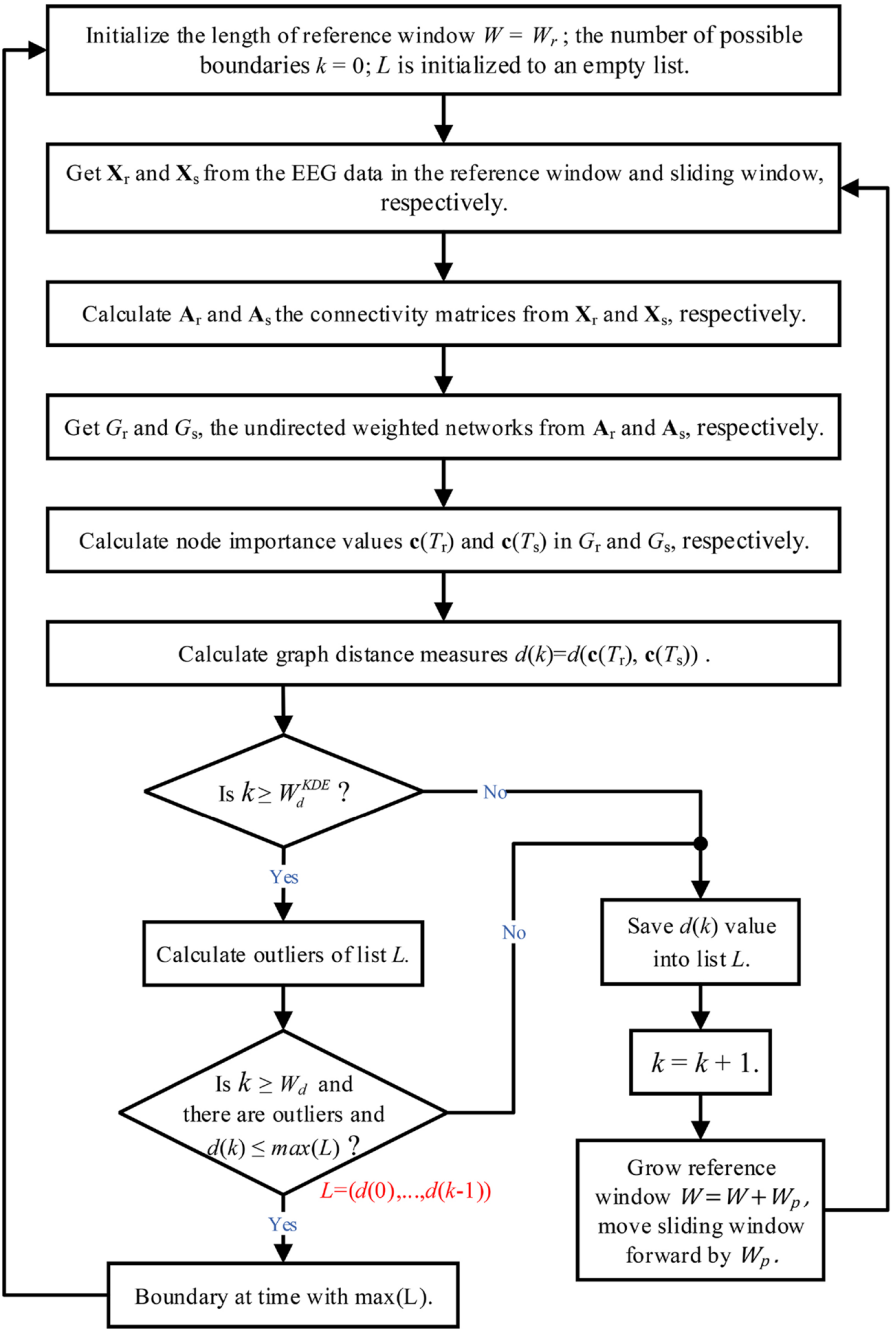
Flowchart of the segmentation algorithm.

To perform temporal segmentation of multi-channel EEG data, we designed a sliding window change detection method based on a previously published method^23^ that compares a sliding window (representing new data) with a growing reference window (Fig. 3, for differences from their algorithm, see the “Discussion”). The parameters of the algorithm are listed in Table 2 (for how these are set, see “Method parameters and evaluation”, a brief discussion of the two most important parameters is in the “Discussion”). In brief, the algorithm calculates the distance between the network in the reference window and that in the sliding window; if this distance is abnormally large (an outlier among samples of distance values), a boundary is declared. The algorithm steps are as follows:

(i) Initialize: length of the reference window $W=W_r$. Number of possible boundaries $k=0$. List of distance values $L=\{\}$. Start of reference window $t_i$ is set to the boundary detected previously (or start of data). The reference window starts from time $t_i$ and goes to time $t_i+W_r$. The sliding window starts from time $t_i+W_r-W_v$ and goes to time $t_i+W_r-W_v+W_s$. The time periods of the reference window and the sliding window are denoted as $T_r$ and $T_s$, respectively.
(ii) EEG data in the reference window is placed in ${\textbf{X}_r}$. EEG data in the sliding window is placed in ${\textbf{X}_s}$.
(iii) Functional connectivity matrices of ${\textbf{X}_r}$ and ${\textbf{X}_s}$ are calculated (with the chosen connectivity measure, e.g. correlation) and stored in ${\textbf{A}_r}$ and ${\textbf{A}_s}$, respectively.
(iv) Undirected weighted networks $G_r$ and $G_s$ are constructed from ${\textbf{A}_r}$ and ${\textbf{A}_s}$, respectively.
(v) Node importance values for each node in $G_r$ and $G_s$ are calculated with the chosen node importance index (e.g. closeness centrality), giving $\mathbf{{c}}(T_r)$ and $\mathbf{{c}}(T_s)$, respectively.
(vi) Graph distance measure *d*(*k*) is calculated by equation (1) with $d(\mathbf{{c}}(T_r), \mathbf{{c}}(T_s))$, i.e. the L2 distance between the node importance value vectors.
(vii) Since we detect change by detecting outliers, a sufficient sample size is necessary to obtain reliable distributions for outlier detection. Hence, we set this criterion: If *k* is less than $W^{KDE}_d$, then (not enough data): *k* is increased by 1 and *d*(*k*) is appended to list *L*; $t_i+W-W_v$ is the time corresponding to this *d*(*k*) entry in *L*; the reference window grows by $W_p$, i.e. its length becomes $W = W+W_p$, while its starting time does not change; the sliding window slides forward in time by $W_p$; go to step ii.
(viii) Outliers of list *L* are calculated (see “Outlier detection”).
(ix) To declare a boundary, an outlier must have been found. *k* must also be at least the required number of comparisons $W_d$. Lastly, the distance *d*(*k*) to the current sliding window should be no greater than the detected outlier in *L*. Hence, we check for these three criteria: (1) outliers in *L* exist; (2) $k \ge W_d$; (3) $d(k) \le max(L)$. If they are all true, we detect a boundary (the largest outlier’s corresponding time is the detected boundary time) and go to step i.
(x) *d*(*k*) is appended to list *L*; the $t_i+W-W_v$ is the time corresponding to this *d*(*k*) entry in *L*; *k* is increased by 1; the reference window grows by $W_p$; the sliding window slides forward in time by $W_p$; go to step ii.

#### Outlier detection

Outlier detection is a key step in the segmentation algorithm: it finds time points with larger than expected graph distances between windows. Considering that the values in list *L* may not be from a normal distribution, we use a non-parametric method for testing outliers. First, we use kernel density estimation (KDE) to obtain a density function. Then a cumulative distribution function *P*(*d*) is estimated. Let $P(d<d_{threshold})=P_{KDE}$, and the threshold $d_{threshold}$ can then be obtained. $P_{KDE}$ is a parameter that specifies the percentile threshold for outliers. All elements of list *L* greater than $d_{threshold}$ are outliers.

To verify the necessity of using a non-parametric method to detect outliers, we performed normal distribution tests for *L* (computed using GD) on real EEG data from 28 participants. Every time outlier detection was required, a normal distribution test was performed. The null hypothesis of normal distribution (significance level 0.05) was rejected on average (across participants) 35.3% of the time.

**Table 2:** Segmentation algorithm parameters.

Parameter	Description
$W_r$	Initial length of the reference window
$W_d$	Minimum number of comparisons before possibly declaring a boundary
$W^{KDE}_d$	Number of comparisons before starting outlier calculation ($W^{KDE}_d\le W_d$)
$W_s$	Length of the sliding window
$W_p$	Increment of the sliding and reference windows
$W_v$	Overlap between sliding window and reference window

## Comparison algorithms

To compare against our proposed framework, we plug in (dis)similarity measures proposed in previous work^14,23^. While their original algorithms work on raw data matrices, we modify them to work on functional connectivity matrices in the interest of fairness, as our simulations focus on functional connectivity changes. Below we present the connectivity-matrix-based (dis)similarity measures used for comparison. In contrast to our proposed method, these approaches focus more on the difference in connection values, rather than graph structure. The EEG data matrix based methods for segmentation^14,23^ use the Kolmogorov–Smirnov (KS) test and the Grassmann distance. The KS test and Grassmann distance are used here on two functional connectivity matrices, instead of two data matrices.

For both the KS and Grassmann approaches, the connectivity matrices $W(T_1)$ and $W(T_2)$ and their eigen-decomposition are calculated to determine their column space and weights of corresponding eigenvectors. The significant eigenvectors are selected by the elbow method to construct feature subspaces $F(T_1)$ and $F(T_2)$, respectively. The Grassmann distance between subspaces is calculated as follows:

2 $$\begin{aligned} d(F(T_1), F(T_2)) = \frac{1}{k} \sqrt{\sum \nolimits _{i = 1}^k \theta ^2_i(F(T_1), F(T_2))}, \end{aligned}$$

where $k = min(dim(F(T_1)), dim(F(T_2)))$, $dim(\cdot )$ is the dimension of a subspace. $\theta _i(F(T_1), F(T_2))$ denotes the *i*th principal angle between subspaces. For the KS test^23^, each column of $W(T_1)$ and $W(T_2)$ is projected onto the feature subspace $F(T_1)$ of the reference window, and the residual of the projections are gathered into two error matrices denoted $e(T_1)$ and $e(T_2)$, respectively. Then, the Kolmogorov–Smirnov test is used to test the difference between $e(T_1)$ and $e(T_2)$, and the final similarity measure is the test’s p-value. A flowchart of the matrix comparison process is shown in Fig. 2B.

In the sliding-window change-detection algorithm, the changes needed for KS and Grassmann approaches are as follows. Grassmann distance: In steps iv–vi, feature subspaces of the matrices $\textbf{A}_r$ and $\textbf{A}_s$ are obtained and Grassmann distance *d*(*k*) is calculated by Eq. (2). Kolmogorov–Smirnov (KS) test: In steps iv–vi, matrices $\textbf{A}_r$ and $\textbf{A}_s$ are projected onto the feature subspace of $\textbf{A}_r$. The residuals after projection are denoted $e(T_r)$ and $e(T_s)$, respectively. The p-value of the KS test between $e_r$ and $e_s$ is stored in *d*(*k*). In outlier detection for the KS test, the smaller the p-value, the greater the difference between two matrices. Thus, in step ix: the condition is reversed, i.e. *d*(*k*) of window *k* is greater than the minimum value of the list *L*. The minimum value in the list *L* is used as the detection boundary. The threshold $d_{threshold}$ is obtained using the cumulative distribution probability $P(d<d_{threshold})=1-P_{KDE}$.

### Ethics statement

The studies involving human participants were reviewed and approved by the institutional human research ethics review committee of the State Key Laboratory of Neuroscience and Learning at Beijing Normal University (ID number: CNL_A_0010_002, approval date November, 2019). The participants provided their written informed consent to participate in this study.

## Simulation results

We first test our segmentation method on simulated EEG data where ground truth of the dynamics of functional connectivity is known. The purpose of this test is to verify that the segmentation method finds the time points where connectivity structure changed. We also vary the difficulty (i.e. magnitude of changes) and examine the effects on accuracy of finding change points. We compare our proposed method with that based on connectivity matrix (edge weight) differences.

### Simulation design

In this section, our segmentation method is evaluated on simulated EEG data. We simulated EEG data with $N=32$ channels, $T=60$s time length, $fs=100$ Hz sampling frequency, and a total of $S=200$ repetitions. The EEG simulation process consisted of first generating several different functional connectivity networks (so that switching among them gives dynamic network structure) , i.e. specifying target connectivity matrices and then generating time-varying EEG data with the target connectivity matrices. We simulated two kinds of changes in network structure, as follows:

**Figure 4:**
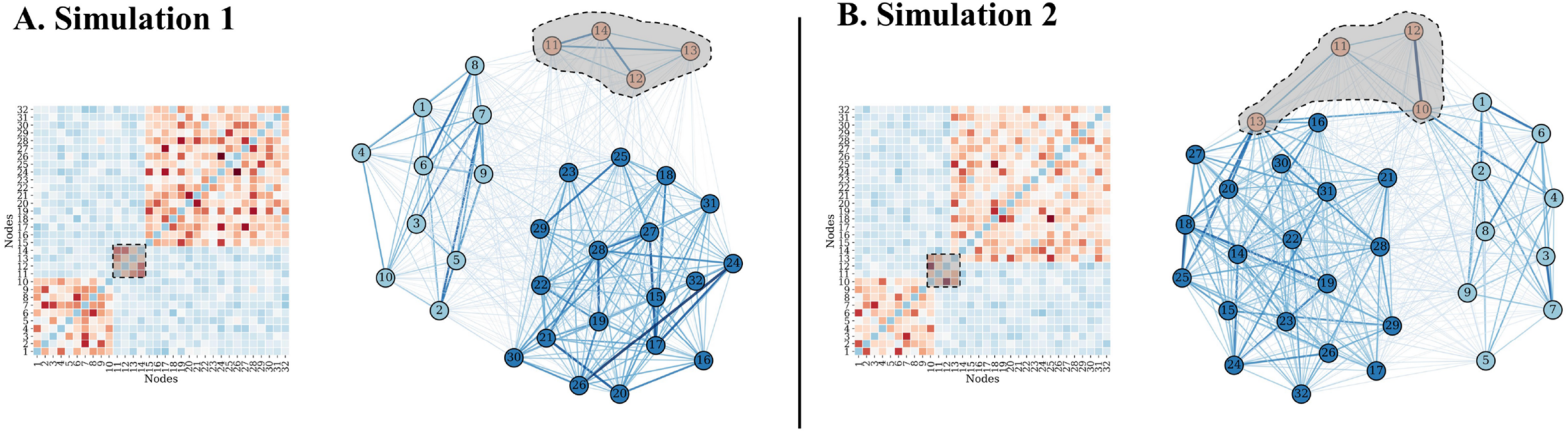
Illustration depicts changes in community structure in the two simulations. (**A**) Conceptual diagram of community changes in Simulation 1, wherein a new community appears (as shown) and subsequently disappears. Left side shows weighted adjacency matrix; right side shows corresponding weighted network. Dashed lines indicate the regions where changes have occurred. Width and color of edges in the network are determined by weight values. Communities are distinguished by the color of nodes. (**B**) Conceptual diagram of changes in Simulation 2, where a central hub appears (as shown) and subsequently disappears. Note that the central hub differs from a regular community in that it has one node which is well-connected to each community.

#### Simulation 1: dynamic network with changing communities

In this simulation, the number of communities changed from 2 to 3 and back. In the interval [1, 20] seconds, the functional connectivity network had two community structures. Community 1 consisted of nodes 1–10 and Community 2 consisted of nodes 15-32. The strength of the connections within the communities was higher (drawn from a normal distribution *N*(0.2, 0.1)) than that between the communities (drawn from *N*(0, 0.05)). To add random diversity, 22 edges and 76 edges were selected from Community 1 and Community 2, respectively, and random weights (*N*(0.2, 0.1)) were added to the original edge weights (the R-MAT algorithm^39^, one of the most commonly-used network generation models; it models graph structure by a degree distribution that follows a power-law distribution, approximating the properties of real-world networks; R-MAT has been previously applied in simulating dynamic brain networks^14^.). The network in the interval [40, 60] s is the same as that in the interval [1, 20] s.

In the interval [20, 40] s, the functional connectivity network had three community structures. Community 1 and Community 2 were the same as in the interval [1, 20] s. Community 3 consisted of nodes 11–14. Weights of the edges within Community 3 were drawn from *N*(0.2, 0.1), and the edges connecting to other communities were drawn from *N*(0, 0.05). According to the R-MAT algorithm, 4 edges in Community 3 were selected to add random weights ($N(0.2k_s, 0.1)$), where the signal strength parameter $k_s$ ranged from [0, 1]. There is a proportional relationship between the $k_s$ value and the signal-to-noise ratio (SNR), as shown in Table 3. The calculation of the SNR is based on the connectivity matrix using a previously published method^14^. The larger $k_s$, the greater the change of the connectivity matrix, and the greater the SNR. The configuration of the connectivity matrix and its corresponding network are illustrated in Fig. 4A.

#### Simulation 2: dynamic network with central hub

Central hubs are a key feature of human brain structural organization, play an important role in function^40^, are involved in changes in brain networks^41,42^, may be useful in decoding brain activity^43^, and may dynamically alternate^44^. Considering their importance, we simulated EEG data where a central hub connecting communities appeared and disappeared.

In the interval [1, 20] s, the connectivity network was the same as that in the interval [1, 20] s of the previous simulation. The difference from Simulation 1 is that Community 2 is set to nodes 13–32. The number of additional weighted edges within Community 2 was changed from 76 to 95. Between [20, 40] s, the network had three communities: Community 1 consisted of nodes 1–10, Community 2 consisted of nodes 13–32, and Community 3 consisted of nodes 10–13. Note that the overlapping nodes make Community 3 a central hub connecting Community 1 and Community 2. The weights of Community 1 and Community 2 were the same as in the interval [1, 20] s. The weights within Community 3 were drawn from *N*(0.2, 0.1), and the edges connected to other communities were drawn from *N*(0, 0.05). Four edges within Community 3 were selected and random weights were added using the R-MAT algorithm. The randomly added weights were drawn from $N(0.2k_s, 0.1)$, where the range of the signal strength parameter $k_s$ was [0, 1]. The configuration of the connectivity matrix and its corresponding network are illustrated in Fig. 4B.

#### Time-varying EEG data generation

There are many methods to simulate time-varying EEG data. For example, the SEED-G toolbox^45^ can simulate EEG data with preset phase-locking values (PLV), phase lag index (PLI), and directional phase lag index (dPLI). However, EEG data with correlation between more than 3 channels cannot be generated due to high computational memory load. The SimMEEG toolbox^46^ can set the number of network nodes (channels), connection strength, network density, and other parameters, but it cannot simulate EEG data with community structure. Šverko^47^ proposed a simulation method that can generate EEG data with community structure, but the community structure is not stable enough over time and connections between communities cannot be preset. Moiseev^48^ proposed a simulation method that allows specified Pearson correlation between channels. Considering computational load and stability of the resulting correlation matrices, we use this method. In this method of generating EEG signals, time courses are generated to have user-specified mutual correlations, and generated signals consist of a combination of evoked signals and (randomized) oscillatory signals. To meet the method’s requirement for positive definite connectivity matrices, we use an iterative spectral method to bend non-positive-definite symmetric matrices to be positive-definite^49^. The bending has little effect on the connectivity matrix weights (Supplementary Figure S1).

To make the properties of the Pearson correlation connectivity matrix closer to those of real EEG data, the connectivity matrices generated in the previous sections need to be adjusted. These adjustments do not change the network structure. First, we normalize: given a connectivity matrix $\mathbf{{W}}$ of size $n \times n$, the connectivity matrix is normalized so all values are [0, 1]: $w^{\prime }_{ij}=(w_{ij} - min(w_{ij}))/(max(w_{ij})-min(w_{ij})), i, j =1,2, \ldots ,n$. Second, we adjust the magnitude of weights, since in real data, Pearson correlation coefficients are not particularly large. We reduce the correlation coefficients in the simulations by multiplying by 0.6^50^, resulting in values in [0, 0.6]. Third, the connectivity matrix is symmetrized: $w^{\prime }_{ij}=w^{\prime }_{ji}, i, j =1,2, \ldots ,n$. Fourth, the diagonals of the matrix are changed to 1: $w^{\prime }_{ij}=1, i=j$.

#### Method parameters and evaluation

The parameters of the segmentation algorithm need to be set to the same values across compared algorithms to ensure fair comparison. We first determined the sensitivity of parameters (i.e. which parameters have a large impact on segmentation results) and found that $W_r$ and $P_{KDE}$ have a relatively large influence on segmentation performance. Thus, it was necessary to perform a grid search for the optimal values of these two parameters (Supplementary Figure S2). For the relatively insensitive parameters, we set $W_s = 2$s, $W_v=1$s, and $W_d=W_d^{KDE}=15$.

To evaluate the performance of each distance measure when combined with the segmentation method, we use several metrics^23^, as follows. Success rate ($p_{succ}$): ratio of number of successfully detected boundaries to the total number of ground truth boundaries. As the length of the sliding window was 2 s, if the detection boundary was within 1 s of the real boundary distance, it was considered a successful detection. Failure rate ($p_{fail}$): ratio of number of falsely detected boundaries to the total number of ground truth boundaries (s). Aggregate rate ($p_{aggr}$): the difference between success rate and failure rate. Average estimated displacement ($\mu _{ED}$): mean of the absolute distance between the detected boundary and nearest ground truth boundary. Here we include all declared detections. The standard deviation of estimation displacement ($\sigma _{ED}$): variability in the displacement around the average displacement $\mu _{ED}$.

### Segmentation results

Eight measures (six choices of node importance indices describing graph structure for our proposed method, two choices of matrix difference measures representing previous work) were plugged into the segmentation framework and tested on the simulated EEG data. Among these, GD and KS are matrix-based measures which represent the traditional approach to segmentation. We also evaluated the Source-Informed Segmentation (SIS) method^23^ in preliminary analysis, but performance on our simulated data was poor (results not shown).

**Figure 5:**
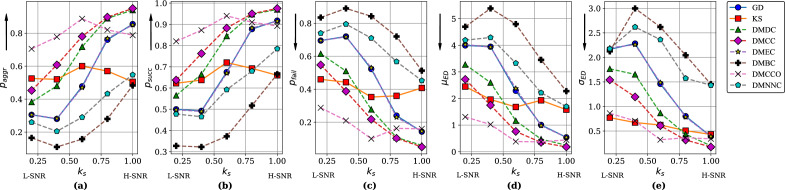
Simulation 1, dynamic network with changing communities: (**a**) aggregate rate ($p_{aggr}$), (**b**) success rate ($p_{succ}$), (**c**) failure rate ($p_{fail}$), (**d**) average boundary displacement ($\mu _{ED}$), and (**e**) standard deviation of displacement ($\sigma _{ED}$) for different signal strength $k_s$. Dotted lines: graph distance measures (proposed). Solid lines: matrix measures (traditional). Y-axis arrows indicate direction of better performance. *L-SNR* lower SNR, *H-SNR* higher SNR.

**Figure 6:**
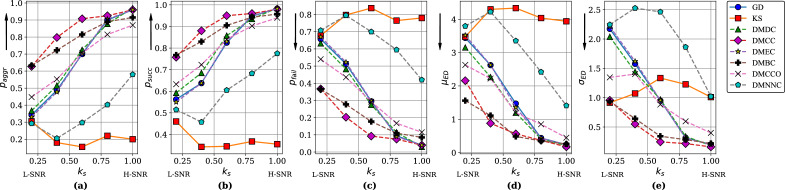
Simulation 2, dynamic network with central hub: (**a**) aggregate rate ($p_{aggr}$), (**b**) success rate ($p_{succ}$), (**c**) failure rate ($p_{fail}$), (**d**) average boundary displacement ($\mu _{ED}$), and (**e**) standard deviation of displacement ($\sigma _{ED}$) for different signal strength $k_s$. Symbols same as above.

The aggregate rate, the success rate, the failure rate, and the average and standard deviation of the boundary displacement at different $k_s$ values are shown in Figs. 5 and 6. From the overall trends we can see that, with an increase of signal strength $k_s$ (the “magnitude” of changes), the performance of distance measures other than KS gradually improved. In Simulation 1, DMCC and DMDC substantially outperformed GD and KS, the two traditional, matrix-based measures. This indicates that with these two node importance indices, network-structure based segmentation (using indices related to graph structure) performs better than functional connectivity matrix based segmentation (directly comparing the edge weights).

When $k_s$ was low, KS and DMCCO also performed well, but segmentation performance did not increase much with $k_s$. In Simulation 2, DMCC and DMBC were better than GD and KS, especially when signal strength $k_s$ was low. DMDC performed slightly better than GD. This again indicates network-structure based segmentation performs better than matrix based segmentation. KS performed poorly in this simulation. The curves for DMEC and GD were found to overlap for both simulations. This is because DMEC and GD are both calculated based on eigenvectors.

To give specific numbers to make the results more intuitive, in Simulation 1, at SNR of about 3dB (signal about twice as large as noise), the DMCC method had a success rate of finding change points of around 88%, with mean error about 0.8 seconds. This shows the segmentation was successful at this SNR level. When SNR was − 1.47 dB (signal size about 0.71 that of noise), DMCC’s accuracy drops to about 64%, with mean error of about 2.8 s.

Overall, these results show that segmentation based on graph distance measures DMCC and DMDC are better than the matrix-based GD and KS measures, in terms of success rate of finding change points and errors in the times of change points detected; these results, together with the relatively poor performance of the SIS method (a method for segmentation that works directly on the data matrices), give support to the proposed approach of segmentation based on functional connectivity structure. In addition, we have explored alternative simulation scenarios, including channel noise, the durations of segments, number and size of network communities. Details of the simulations and results can be found in the Supplementary Materials, particularly Figure S3. In summary, the results indicate that our proposed method performed better than GD and KS over a wide range of simulation parameters.

**Table 3:** SNR corresponding to signal strength $k_s$ values.

Signal strength $k_s$	0.2	0.4	0.6	0.8	1.0
Sim 1 SNR (dB)	− 1.47	1.00	3.07	4.46	5.91
Sim 2 SNR (dB)	− 1.55	0.36	2.35	3.98	5.26

### Computational cost

**Figure 7:**
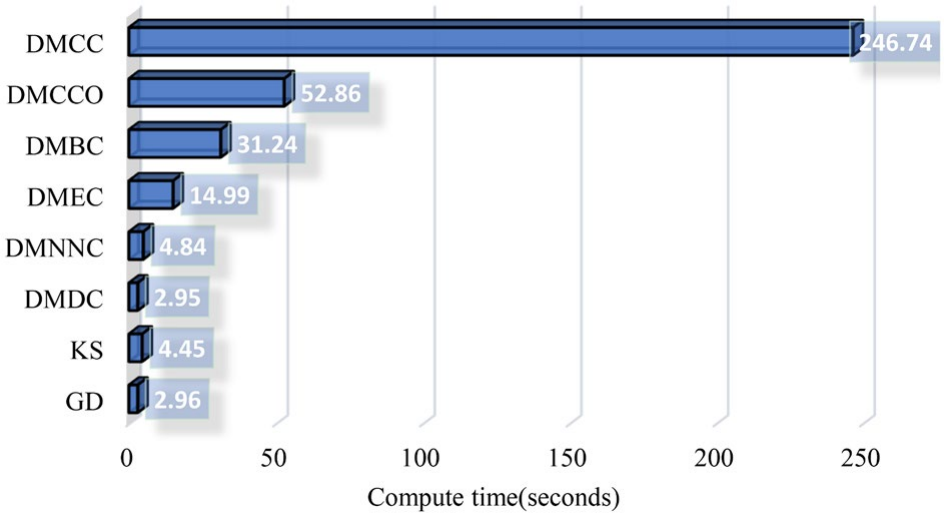
Computational costs for the graph distance and matrix measures. DMDC, DMCC, DMEC, DMBC, DMCCO, and DMNNC denote the 6 types of graph distance measures (Table 1). GD and KS denote Grassmann distance and Kolmogorov–Smirnov test, respectively. Times shown are for analyzing 10 one-minute portions of 32-channel EEG data on a Core i7-10700.

Here we compare the computational costs (actual run times) of the six node importance indices and the two matrix-based difference measures. We implemented the segmentation algorithm in Python 3.8.8 on a desktop computer with a Windows 11 64-bit operating system, 16 GB DDR4 memory, and Intel Core i7-10700 2.90 GHz 16-core processor. Segmentation run times were obtained by processing 10 one-minute portions of simulated data with parameter settings as above. The wall-clock execution times are shown in Fig. 7. The computational costs of GD, KS, DMDC, and DMNNC were similar and relatively low. The computational costs of DMCC, DMCCO, DMBC, and DMEC were relatively high, due to the complexity involved in calculating node centrality, particularly for DMCC, which requires computation of shortest paths (Dijkstra’s algorithm). These results show that while DMCC performs well in the simulations, it has relatively large computational cost. DMDC, which also performs well, but not quite as well as DMCC overall, has low computational cost, making it a priority candidate when computational resources are limited.

## Real EEG data results

In this section, we use real EEG data to examine the performance of the proposed segmentation algorithm and compare the six node importance indices, as well as the matrix-based approach. The purpose of this portion of the study is to show that the method can work on real EEG data better than comparison methods. Our EEG data comes from a study geared towards real-world application, in which recordings were made with a small number of electrodes to mimic practical recording hardware; but this means source localization and source connectivity analysis is impractical, so we use electrodes as network nodes. The following describes the process of data acquisition, the evaluation procedure, and the segmentation results.

### EEG data and pre-processing

EEG data was collected from thirty-one college students (15 females and 16 males; 22.4 ± 2.7 years age, right-handed; three participants’ data were not included in the analysis due to data deficiency)^51^. An ethics statement regarding this experiment is described at the end of this article.

Each participant controlled a virtual drone to hit floating balloons in a flight simulator. The task had two difficulty levels: easy and difficult. The balloons were more densely distributed in the easy condition compared to the difficult condition. The duration of each condition was about 5 minutes. All participants completed the easy condition first, followed by the difficult condition, with a short break in between. To study event-related potentials and mental workload, the participants were stimulated with auditory beeps during the experiment, and the interval between stimuli was randomly chosen between 1.5 and 5 s.

The raw data were first down-sampled to 256 Hz and then notch filtered (48–52 Hz) to remove power-line noise. EEG signals were then band-pass filtered using a windowed-sinc finite impulse response filter (kernel order automatically chosen by the pop_EEGfiltnew function of EEGlab) into the gamma frequency band (30–80 Hz).

### Evaluation procedure

**Figure 8:**
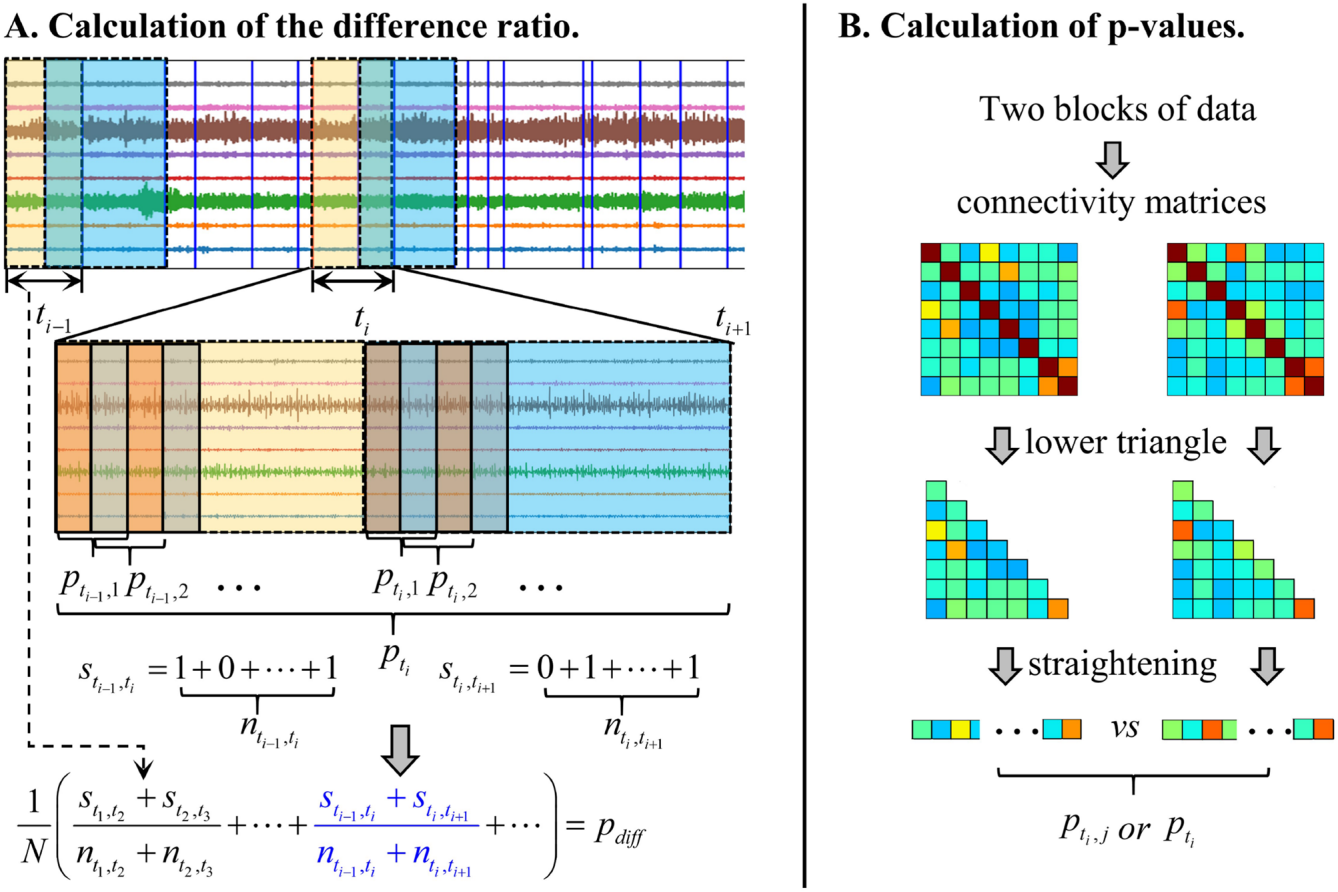
Schematic diagram of the calculation of the difference ratio $p_{diff}$. (**A**) the calculation process of the difference ratio, (**B**) the calculation of the p-value between two segments of EEG data.

Considering that real EEG data do not come with ground truth boundaries, the measures of segmentation performance used in the previous section cannot be used here. Generally, segmentation of EEG data is to ensure that data in a segment have quasi-stationary statistical properties, and the data of different segments have large statistical differences. Therefore, we designed an index to measure the relative size of inter-segment differences compared to intra-segment differences:

Let the EEG data be stored in the matrix $\textbf{X}_{n \times T}$, *n* is the number of channels, and *T* is the time. Data $\textbf{X}$ are divided into *N+1* intervals. The set $L_{inter}=\{t_i, i=1, 2, \ldots, N+2 \}$ is composed of detected boundary time points, and $t_i$ is a boundary time point. A connectivity matrix is constructed for the data in interval $[t_{i-1}, t_i]$. Here we consider the symmetric connectivity matrix and straighten the upper triangular elements of the matrix to obtain the vector $v_{i-1}$. Similarly, the connectivity matrix generated by the data in the interval $[t_i, t_{i+1}]$ is straightened into vector $v_{i}$. A Wilcoxon signed-rank test^52^ is performed for vectors $v_{i-1}$ and $v_{i}$, yielding a p-value $p_{t_i}$. The p-value gives the probability of falsely rejecting the null hypothesis that the two vectors come from the same distribution. The smaller the p-value, the more convincing the differences in the connectivity matrices. The interval $[t_{i-1}, t_i]$ is then divided into $n_{t_{i-1}, t_i}+1$ smaller time segments of fixed length *Ls*. The time points of these smaller segments form the set $L_{inter,t_{i-1}}=\{ t_{i-1, j}, j=1, 2, \ldots , n_{t_{i-1}, t_i}+2 \}$. As, the data interval $[t_{i-1}, t_i]$ is generally not divisible by *Ls*, there will be a remainder at the end, which we ignore, as this segment is relatively small compared to $[t_{i-1}, t_i]$. From adjacent small segments $[t_{i-1, j-1}, t_{i-1, j}]$ and $[t_{i-1, j}, t_{i-1, j+1}]$ we construct two symmetric connectivity matrices. The upper triangle of these two matrices are straightened into two vectors $v_{t_{i-1, j-1}}$ and $v_{t_{i-1, j}}$, respectively. Then the p-value $p_{t_{i-1}, j}$ of the Wilcoxon signed-rank test between these vectors is calculated. Repeating this process for the other pairs of neighboring small segments, we obtain $n_{t_{i-1}, t_i}$ p-values from the interval $[t_{i-1}, t_i]$. We also obtain $n_{t_i, t_{i+1}}$ p-values from the interval $[t_{i}, t_{i+1}]$. These p-values can be compared due to equal sample sizes^53^. A diagram of this p-value calculation is shown in Fig. 8B.

Let $s_{t_{i-1}, t_i}$ be the number of $p_{t_i-1, j}$ larger than $p_{t_i}$, i.e. the number of intra-interval differences smaller than the inter-interval difference. The formula for $s_{t_{i-1}, t_i}$ is as follows:

3 $$\begin{aligned} s_{t_{i-1}, t_i} = \sum \nolimits ^{n_{t_{i-1}+1, t_i}}_{j=2} {\delta ({p_{{t_{i - 1}},j}})} \end{aligned}$$

where the function $\delta (\cdot )$ is

$$\begin{aligned} {\begin{matrix} \delta ({p_{{t_{i - 1}},j}}) = \left\{ \begin{array}{lr} 1&{}{{p_{{t_{i - 1}},j}} > {p_{{t_i}}}} \\ 0&{}{{p_{{t_{i - 1}},j}} \le {p_{{t_i}}}} \end{array} \right. \end{matrix}} \end{aligned}$$

Similarly, let $s_{t_i, t_{i+1}}$ be the number of $p_{t_i, j}$ larger than $p_{t_i}$. We define a difference ratio as follows:

4 $$\begin{aligned} p_{diff} = \frac{1}{N} \sum \nolimits _{t_i,i=2,...,N+1} \frac{s_{t_{i - 1},t_i} + s_{t_i,t_{i + 1}}}{n_{t_{i - 1},t_i} + n_{t_i,t_{i + 1}}} \end{aligned}$$

The higher the $p_{diff}$ value, the better the segmentation performance. The calculation process of $p_{diff}$ is shown in Fig. 8A.

Since the easy condition and difficult condition were performed in two blocks with a rest in between, we segmented data from the two conditions separately. In the experiment, there were sound stimuli (SS) at random times, which produced event-related potentials in the EEG data. Therefore, we also used the sound stimulation times as segment boundaries for comparison. We also added a naive segmentation method for comparison, where each segment was the same length (SL), 800 samples (for a similar total number of segments as SS).

We repeated segmentation with Pearson correlation (PC) and phase locking value (PLV) as two choices for the connectivity matrix calculation method. The parameters of the segmentation algorithm were set as: $W_d = W^{KDE}_d = 30$, $W_r = W_s=2$s, $W_v =1$s, $Wp = 10$, $p_{KDE} = 0.985$. The parameters $(W_r, p_{KDE})$ had a relatively large effect on $p_{diff}$ and the number of segments found. For these two parameters, we did a grid search, and the results for GD and DMDC are shown in Supplementary Figures S4 and S5 (PC was used here for connectivity calculation). Other segmentation methods had similar results for the grid search on $(W_r, p_{KDE})$. Since future application of our method involves studying dynamic changes in the functional connectivity networks and using network features as inputs for neural decoding, the number of segments should not be too small. We also chose parameters so that the number of detected segments is similar to the number of sound stimuli, to have comparable $p_{diff}$ values.

### Segmentation results

**Figure 9:**
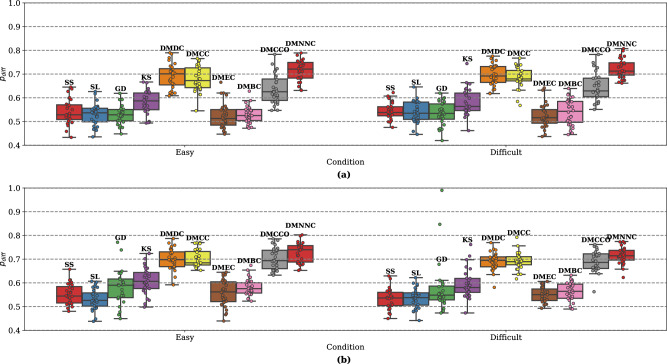
Segmentation performance as measured with $p_{diff}$ on real EEG data (higher values are better). (**a**) connectivity calculated by Pearson correlation, (**b**) phase-locking value. SS denotes segments with boundaries at sound stimulation times. SL denotes segmentation into same-length time windows.

The EEG data of 28 participants were segmented and the difference ratio $p_{diff}$ values are shown in Fig. 9. For both Pearson correlation (Fig. 9a) and phase-locking value (Fig. 9b) connectivity matrices, graph distance measures DMDC, DMCC, DMNNC, and DMCCO performed better than the other segmentation methods in terms of $p_{diff}$, meaning that intra-segment differences were smaller than inter-segment differences. The segmentation based on sound stimulation (SS) performed poorly, suggesting that the sound stimulus onset times did not mark boundaries in network structure change. This concurred with previous decoding analysis results on this data, which found that windows around the sound stimulation times were not the best choice of epochs for mental workload classification^51^. For the same length window segmentation, network structure between segments was not significantly different, showing that this naive approach is inappropriate. For GD, data from 4 participants’ easy condition were not segmented, and data from 2 participants’ difficult condition were not segmented (i.e. no boundaries were found). These unsegmented participants are not included in Fig. 9, but the numbers of segments are included in Supplementary Figure S6. For GD, the data from the participant with highest $p_{diff}$ in the difficult condition (value near 1.0) were divided into only two segments. The number of segments for each method is detailed in Supplementary Figure S6. From Supplementary Figure S6a and b, we can see that the number of segments found by KS varies greatly among participants, and the number of segments for other methods had smaller variance.

Overall, DMDC, DMCC, DMNNC, and DMCCO outperformed (as in, inter-segment differences were larger than intra-segment differences) the methods based on matrix difference measures (GD and KS), as well as the two naïve methods for segmentation (SS and SL), on real data. These results support the higher effectiveness of the functional connectivity structure approach to segmentation when used on real EEG data.

**Figure 10:**
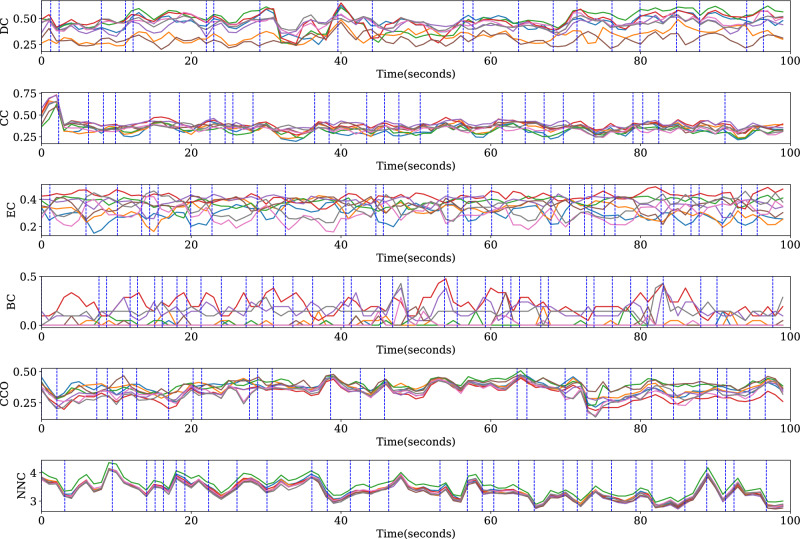
Visualization of results of segmentation based on different node importance indices on networks constructed by Pearson correlation. EEG data and segmentation results of the highest $p_{diff}$ are shown. Curves in the graph show node importance of all nodes, obtained in sliding windows (length 2 s, overlap 1 s). The data of the first 100 s are displayed here. Vertical dashed lines are segment boundaries.

**Figure 11:**
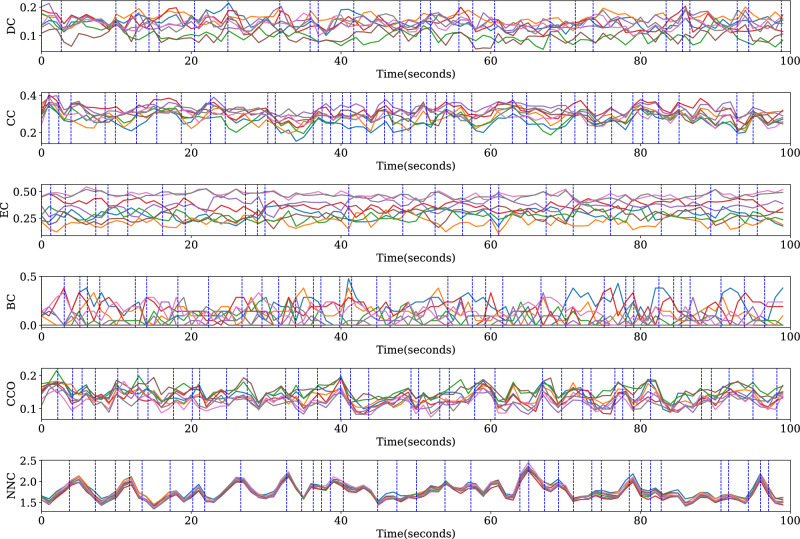
Visualization of results of segmentation based on different node importance indices on networks constructed by PLV. EEG data and segmentation results of the highest $p_{diff}$ are shown. Curves in the graph show node importance of all nodes, obtained in sliding windows (length 2 s, overlap 1 s). The data of the first 100 s are displayed here. Vertical dashed lines are segment boundaries.

In Figs. 10 and 11, we visualize the segmentation performance of the segmentation methods with graph distance measures. EEG data (28 $\times$ 2 data) and segmentation methods corresponding to the highest $p_{diff}$ are selected to visualize. The node importance over time is obtained using the sliding window method. We found that the fluctuations of EC and BC of each channel over time are relatively more disordered, which is not conducive to segmentation, the $p_{diff}$ value using DMEC and DMBC is low in Fig. 9. For other segmentation methods using graph distance measures, the boundary is close to the inflection point on the curve.

## Discussion

In this study, we proposed an EEG segmentation method based on graph distance measures to solve the problem of how to segment EEG data according to changes in the functional connectivity network structure so that within each segment, differences in network structure remain small while between segments, differences are large. We designed a graph distance framework to provide an intuitive scalar measure for differences in network structure. We combine graph distance with a segmentation procedure that looks for abnormally large distances between a sliding window and a reference window. We evaluated our segmentation method on simulated EEG data under two network structure changes. Finally, we segmented real EEG data and quantified the connectivity changes within segments versus between segments.

Although the closeness centrality graph distance measure (DMCC) performed well on both simulated data and actual data, its computational cost was relatively high. The main reason is that the calculation of shortest paths for closeness centrality has high computational cost. We used Dijkstra’s algorithm^54^ to calculate the shortest path between nodes. Considering that our graphs are dense, i.e. we need to traverse all edges, the time complexity of CC is $O(n^3)$, where *n* is the number of EEG channels.

Graph distance measures DMDC and DMCC have relatively good segmentation performance, as seen in Figs. 5, 6, and 9. DMBC performed well on Simulation 2 but poorly on Simulation 1. DMNCC performed poorly on both simulations but performed well on the real data. The six graph distance measures use node indices that ascribe importance to nodes in different ways, which leads to varying sensitivity to different network structures. In real applications, which measure is best suited may be difficult to predict a priori; therefore, we recommend the use of multiple graph distance measures and performing model selection. Generally, there are many indices for importance of nodes, such as Katz’s centrality^55^, PageRank^56^, LeaderRank^57^, and semilocal centrality^58^. The choice of node importance index requires attention to interpretation and computational complexity. We can select the node importance index according to the network structure changes of interest. For example, closeness centrality describes the transmission efficiency of this node (brain region) to all other nodes. Using DMCC, at the time points of segmentation, there are large changes in the transmission efficiency of brain regions. There are some relationships between the six node importance indices. They characterize different aspects of network structure, and the relationship between them thus depends on the specific network characteristics or structure^59^. If there is prior knowledge about the form of the graph of the functional connectivity network, then we can say something more specific, for example, in scale-free networks, DMDC and DMBC are strongly correlated^60^. The flexibility of our approach allows different indices to be tested for an application. How indices relate to each other and the theory behind which index is best for segmentation in a given situation are important questions, which we do not try to answer here; but we have presented empirical comparisons in our simulations and analysis on real EEG data.

In our segmentation procedure, the length of the reference window $W_r$ and the probability $p_{KDE}$ in the detection of outliers have large impacts on the number of segments. The reference window determines the minimum length of detected segments. A shorter reference window can be used if one believes changes in EEG data happen frequently. The larger the probability $p_{KDE}$, the less sensitive the outlier detection, and the number of detected segments will be smaller. When one believes the network structure has slow changes, we recommended a smaller $p_{KDE}$.

The KS matrix measure represents previous work, the Source-Informed Segmentation method (SIS)^23^. Many steps of the presented sliding-window change-detection mechanism are also based on SIS’s sliding-window algorithm. However, there are crucial differences in the overall framework. In the SIS method, the patterns of EEG voltage values of multiple channels (the EEG data matrix) are examined for changes, while in our framework, the differences in a chosen graph measure computed from functional connectivity networks (e.g. connectivity matrices) built from EEG data are examined. Our method also differs in the change detection mechanism: we identify a time window that has a distance value (or p-value) which is an outlier of the reference distribution of distances (p-values). In contrast, SIS^23^ uses the p-values from K–S testing to directly decide significance. Our approach prevents situations where the largest distance (or smallest p-value) is not necessarily significantly higher (lower) than the other distances (p-values).

In Simulations 1 and 2, the GD method performed better than some graph distance measures. Especially in Simulation 2 at higher SNR, GD performed the best. Segmentation based on graph distance measures are relatively more sensitive to small network changes. In addition, the results of GD and DMEC were found to be coincident in the two simulations (Figs. 5, 6). Their results on real data are also similar (Fig. 9). This is likely because they both are calculated by eigenvectors, but we do not have an analytical proof of equality.

There are some limitations to our study. There exist other segmentation methods which we did not compare here. In this study, we only compare to GD and KS (as well as to stimulation-based segmentation and same-length window segmentation), because these also look at differences in functional connectivity, while other methods in the field optimize for other criteria. Each segmentation method has a different definition or goal and thus may be better suited for a particular analytical purpose. Another limitation is that our real data experiments were conducted using electrodes as network nodes, which means computed networks may include artifactual edges due to electrical conduction. However, this noiseness in the data is faced by all methods in our experiments.

We only consider single-layer undirected weighted networks. When there is a negative correlation, we use the absolute value for the weight. As a result, some information will be lost. In follow-up work, negative correlation can be considered and more complex networks can be constructed, such as directed networks or multi-layer networks. We only use one simulation method to make our simulated data, so performance under other simulation methods needs further verification.

We only tested our method with correlation and PLV as the functional connectivity metric, while many other metrics exist. Our framework is compatible with other metrics, but whether our method will work well with other metrics is an open question, and likely depends on the application and data characteristics.

## Conclusion

In this work, we designed a graph distance framework and a segmentation procedure for the segmentation of EEG data. According to our results on simulated EEG data and real EEG data (with correlation or PLV as connectivity metric), the graph distance measures based on degree centrality and closeness centrality outperform matrix-based measures (Grassman distance and Kolmogorov–Smirnov). When also considering computational cost, the degree centrality approach presents a good overall choice. Performance of various node importance indices vary per dataset, suggesting that selection of node importance index is dependent on characteristics of data.

We provide a new perspective for segmentation, one based on network structure changes, and demonstrate its effectiveness with correlation and PLV connectivity, which suggests more segmentation methods based on network structure properties can be investigated. Our results suggest that when using an appropriate graph distance measure for the data, segmentation can be more sensitive and effective than matrix-based segmentation.

### Supplementary Information

Supplementary Information.

## Data Availability

The measurement data supporting the conclusions of this article will be made available by the authors, without undue reservation.
