# Supplementary material for: Temporal segmentation of EEG based on functional connectivity network structure
Source: Sci Rep. 2023 Dec 19;13:22566. doi: 10.1038/s41598-023-49891-8 (PMC10730570; doi:10.1038/s41598-023-49891-8)
Supplement: Supplementary file 1 — Supplementary Information. [file 41598_2023_49891_MOESM1_ESM.pdf]

Supplementary Materials

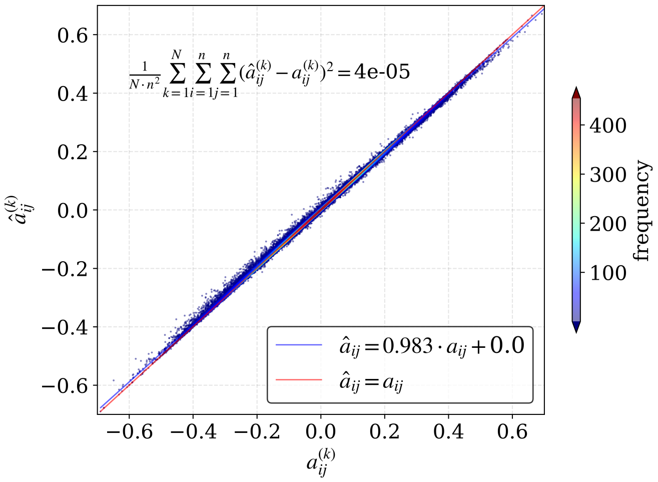

Figure S1. In the simulation generation procedure, the bending procedure to make connectivity matrices positive-definite did not substantially change target connectivity matrix values. Horizontal axis shows entries $a_{ij}^{(k)}$ of the randomly generated target correlation matrix $\mathbf{A}$. Vertical axis shows entries $\hat{a}_{ij}^{(k)}$ after bending to a positive-definite matrix $\hat{\mathbf{A}}$.

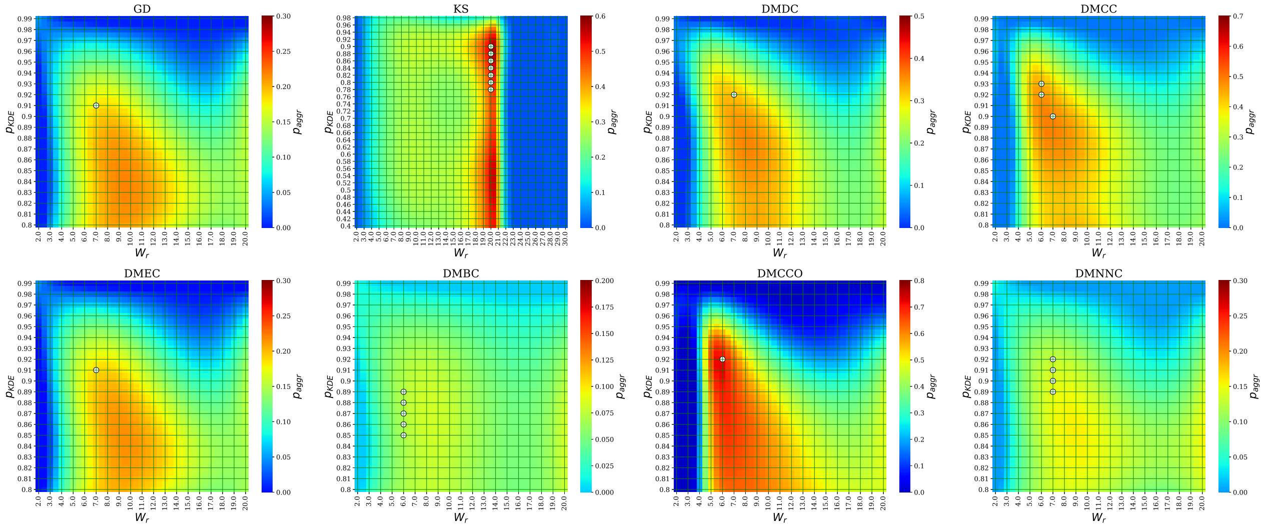

Figure S2. Grid search for reference window length (horizontal axis) and probability threshold for outlier detection (vertical axis). Each subplot shows p_{aggr} (aggregate probability of success, higher the better) for a graph distance or matrix difference measure. Simulations here used $k_s = 0.4$ with scenario from Simulation 1. Global maxima are indicated by circles (there may be ties). Results for other k_s values and Simulation 2 are similar to results here.

Supplementary simulations

We conduct supplementary simulations to investigate the potential influence of channel noise, segment duration, community size, and the number of communities. Here, we keep a fixed k_s value of 0.4, which means the signal-to-noise ratio is approximately constant (except in the channel noise simulations, where the channel noise changes the SNR). In these supplementary simulations, we select the optimal parameter combination for the reference window length W_r and probability P_{KDE} based on the maximum value of aggregate rate p_{aggr}. To limit computational time, and using the knowledge gained from the main simulations about the likely range of optimal parameter values, we set the range of optimization for W_r as [0.85, 95] and for P_{KDE} as [2, 17]. To limit computation time, we repeat the calculations only 20 times during parameter search. Then, once the optimal W_r and P_{KDE} parameters were found, we segment 200 batches of randomly generated data using the optimal parameter set. All other parameters were set to the same values as the main simulations, except those intentionally varied, as described in the following.

Channel noise

The data generation in the main experiments included oscillatory noise, but not Gaussian channel noise. Here we add channel noise with mean of 0 and standard deviation of 1. The noise signal is multiplied by a coefficient k_{noise}, to control the impact of noise on the simulated signal. The range for k_{noise} is between 0 and 0.6. We calculate SNR for the simulated data under different values of k_{noise}, by $SNR = 10 \cdot \log_{10}(Ps/Pn)$, where Ps represents the average power of the simulated signal (excluding channel noise), and Pn represents the average power of the channel noise.

The impact of channel noise on segmentation accuracy (measured by the aggregate rate P_{aggr} metric) is depicted in Figure S3 (a) and (b), corresponding to modified Simulation 1 and 2, respectively. SNR is shown in the x-axis labels in parenthesis. As the channel noise increases, the segmentation results deteriorate. Over various amounts of channel noise in Simulation 1, DMCC performs better than GD and KS (Figure S3 (a)). In Simulation 2, both DMBC and DMCC outperform GD and KS (Figure S3 (b)).

Duration of segments

In the main experiments, the duration for each segment was 20 seconds. Here, we test segment durations of 10, 15, 20, 25, and 30 seconds. The impact of segment duration on the segmentation accuracy is depicted in Figure S3 (c) and (d), corresponding to modified Simulation 1 and 2, respectively. Variation in segment duration had a relatively minor impact on the segmentation accuracy. Over various segment durations for Simulation 1, DMCCO, DMCC, and DMDC performed better than GD and KS (Figure S3 (c)). In Simulation 2, DMCC, DMBC, and DMCCO performed better than GD and KS (Figure S3 (d)).

Size of communities

In the main experiments, the size of Community 3 was 4 nodes. Here, we set the size of Community 3 in Simulation 1 and Simulation 2 to 2, 4, 6, 8, or 10. The impact of community size on the segmentation accuracy is depicted in Figure S3 (e) and (f), corresponding to modified Simulation 1 and 2, respectively. As the changing community and central hub become larger, the segmentation results of all methods improve. For Simulation 1, DMCC and DMDC demonstrate the best performance, better than GD and KS (Figure S3 (e)). For Simulation 2, DMCC performs the best overall, better than GD and KS (except at 8 and 10, where GD performed the best) (Figure S3 (f)).

Number of communities

In the main experiments, the number of communities changes between 2 and 3. Here, we sequentially add changing communities with 4 nodes into the network of Simulation 1. The range for the number of added communities is [1, 5]. Since a central hub is by definition singular, we do not redo Simulation 2 in these supplementary simulations.

The impact of adding communities on the segmentation accuracy is depicted in Figure S3 (g). As the number of changing communities increases, the segmentation accuracy values of all methods improve (Figure S3 (g)). DMCC performs the best, better than GD and KS.

Overall, in simulations varying over the above settings, segmentation methods based on graph distances still demonstrate an advantage over traditional methods GD and KS, supporting the use of functional connectivity structure for segmentation.

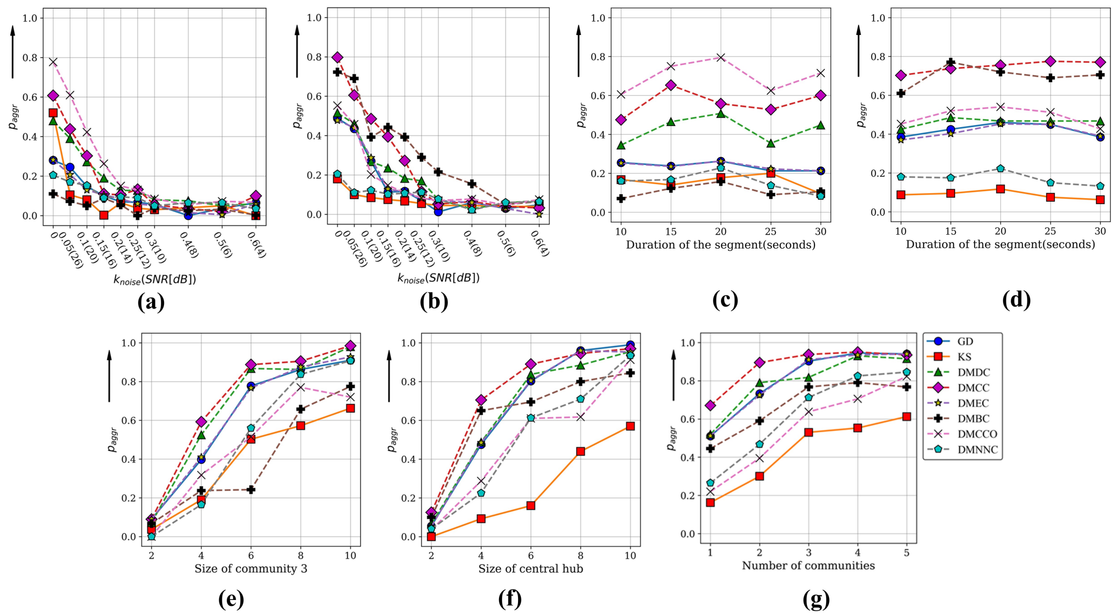

Figure S3. (a) Aggregate rate(p_{aggr}) for different levels of Gaussian channel noise in Simulation 1. (b) Aggregate rate for different levels of Gaussian channel noise in Simulation 2. (c) Aggregate rate for different durations of segments in Simulation 1. (d) Aggregate rate for different durations of segments in Simulation 2. (e) Aggregate rate for different community sizes of Community 3 in Simulation 1. (f) Aggregate rate for different central hub sizes in Simulation 2. (g) Aggregate rate for different number of communities in Simulation 1.

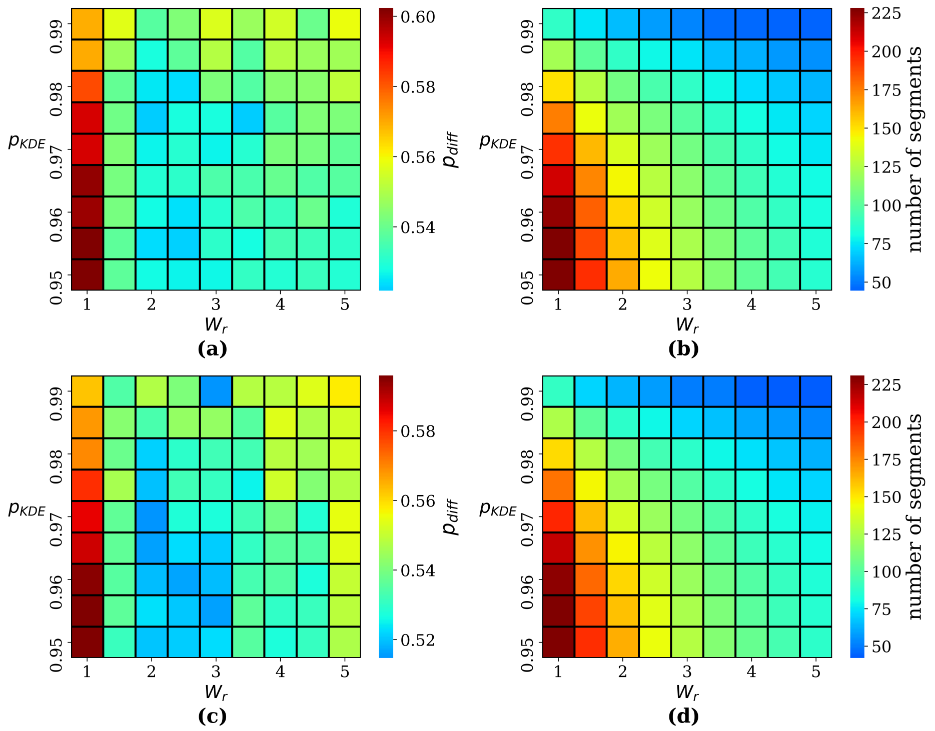

Figure S4. Segmentation performance of GD for different settings of (W_r, p_{KDE}) on real EEG data. (a) difference ratio p_{diff} in easy condition; (b) number of segments in easy condition; (c) difference ratio p_{diff} in difficult condition; (d) number of segments in difficult condition.

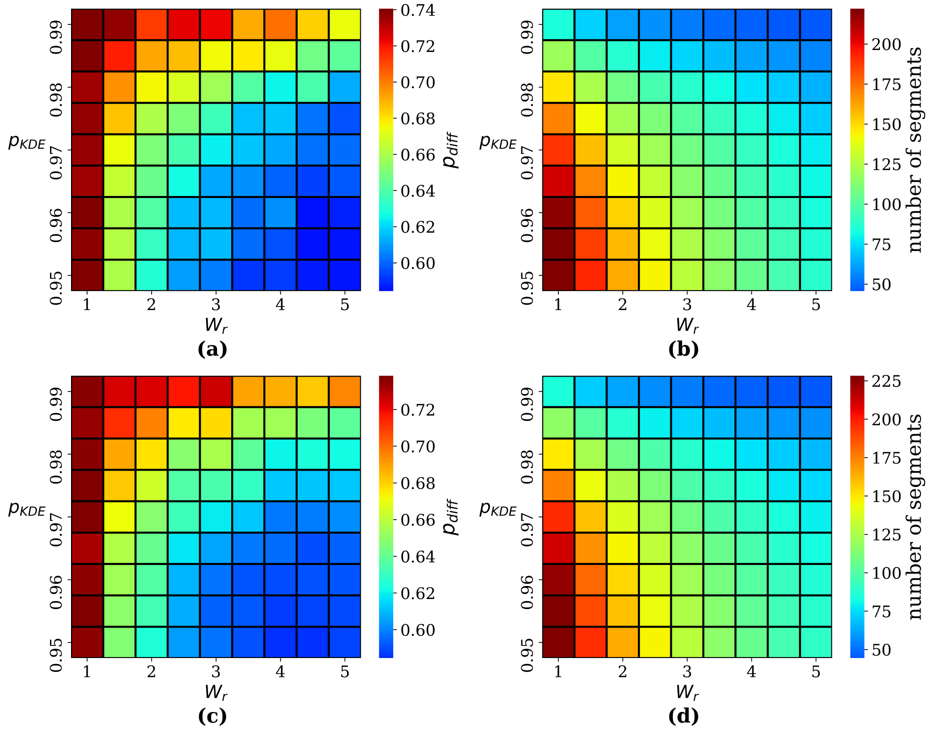

Figure S5. Segmentation performance of DMDC for different settings of (W_r, p_{KDE}) on real EEG data. (a) difference ratio p_{diff} in easy condition; (b) number of segments in easy condition; (c) difference ratio p_{diff} in difficult condition; (d) number of segments in difficult condition.

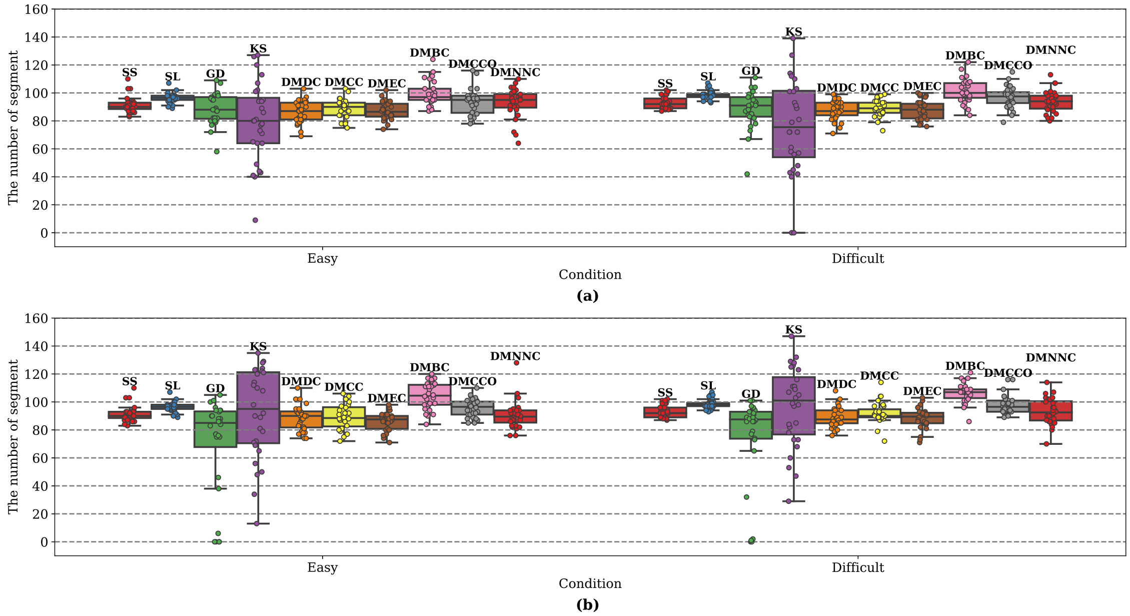

Figure S6. Number of segments found on real EEG data. (a) connectivity calculated by Pearson correlation, (b) phase-locking value. SS denotes segments with boundaries at sound stimulation times (random intervals between 1.5s and 5s). SL denotes segmentation by same-length time windows (800 samples).
